# Experimental transmission of *Anaplasma marginale *by male *Dermacentor reticulatus*

**DOI:** 10.1186/1746-6148-3-32

**Published:** 2007-11-30

**Authors:** Zorica Zivkovic, Ard M Nijhof, José de la Fuente, Katherine M Kocan, Frans Jongejan

**Affiliations:** 1 Utrecht Centre for Tick-borne Diseases (UCTD), Department of Infectious Diseases and Immunology, Faculty of Veterinary Medicine, Utrecht University, Yalelaan 1, 3584CL, Utrecht, The Netherlands; 2 Instituto de Investigación en Recursos Cinegéticos IREC (CSIC-UCLM-JCCM), Ronda de Toledo s/n, 13071 Ciudad Real, Spain; 3 Department of Veterinary Pathobiology, Center for Veterinary Health Sciences, Oklahoma State University, Stillwater, OK 74078, USA; 4 Department of Veterinary Tropical Diseases, Faculty of Veterinary Science, University of Pretoria, Private Bag X04, 0110, Onderstepoort, South Africa

## Abstract

**Background:**

Bovine anaplasmosis has been reported in several European countries, but the vector competency of tick species for *Anaplasma marginale *from these localities has not been determined. Because of the wide distributional range of *Dermacentor reticulatus *within Europe and the major role of *Dermacentor *spp. as a vector of *A. marginale *in the United States, we tested the vector competency of *D. reticulatus *for *A. marginale*.

**Results:**

Male *D. reticulatus *were allowed to feed for 7 days on a calf persistently infected with a Zaria isolate of *A. marginale*, after which they were removed and held off-host for 7 days. The ticks were then allowed to feed a second time for 7 days on a susceptible tick-naïve calf. Infection of calf No. 4291 was detected 20 days post exposure (p.i.) and confirmed by *msp4 *PCR. Thirty percent of the dissected acquisition fed ticks was infected. In addition, *A. marginale *colonies were detected by light microscopy in the salivary glands of the acquisition fed ticks. Transmission of *A. marginale *to calf No. 9191 was confirmed by examination of Giemsa-stained blood smears and *msp4 *PCR. Ticks were dissected after transmission feeding and presence of *A. marginale *was confirmed in 18.5% of the dissected ticks.

**Conclusion:**

This study demonstrates that *D. reticulatus *males are competent vectors of *A. marginale*. Further studies are needed to confirm the vector competency of *D. reticulatus *for other *A. marginale *strains from geographic areas in Europe.

## Background

Bovine anaplasmosis is one of the most important tick-borne diseases of ruminants worldwide. The disease is caused by infection of cattle with the obligate intraerythrocytic bacteria *Anaplasma marginale *which is classified in the family Anaplasmataceae, order Rickettsiales [1]. The acute phase of the bovine anaplasmosis is characterized by anemia, icterus, weight loss, fever, abortion, decreased milk production and often results in death [2]. Animals surviving the acute phase develop a lifelong persistent infection and can serve as reservoirs for mechanical transmission and biological transmission by ticks [3].

Anaplasmosis is endemic in tropical and sub-tropical regions where the disease constitutes a constraint to the cattle production. In Europe anaplasmosis is endemic in several Mediterranean countries including Italy [4,5], Portugal [6] and Spain [7], and has occasionally been reported in Austria [8], Switzerland [9] and Hungary [10]. Mechanical transmission of *A. marginale *is effected by blood-contaminated fomites, including hypodermic needles, castration instruments, ear tagging devices, tattooing instruments, and dehorning saws or by blood-contaminated mouthparts of biting flies [11]. Biological transmission is effected by ticks and over 20 species of ticks have been incriminated as vectors worldwide [12]. While the one-host ticks, *Rhipicephalus *(*Boophilus) microplus *and *R. annulatus*, were eradicated from the United States in the early 1940s, they are the main tick vectors in tropical and subtropical areas [13]. Currently, *Dermacentor *spp. (*D. andersoni*, *D. variabilis *and *D. albipictus*) are the major tick vectors of *A. marginale *in the U.S. [14].

*A. marginale *undergoes a complex developmental cycle in ticks that begins with infection of gut cells from infected erythrocytes ingested with the tick bloodmeal [15,16]. Development of the final infective stage occurs in salivary glands from where the pathogen is transmitted to cattle. A major means of *A. marginale *transmission appears to be by male *Dermacentor *ticks which become persistently infected. These males are intermittent feeders and can feed and transmit *A. marginale *multiple times as they transfer among cattle, thus effecting intrastadial transmission [15,16].

The vectorial capacity of tick species for *A. marginale *in Europe has not been well defined. Recent reports of endemicity of anaplasmosis in European countries [10] and of outbreaks in countries previously thought to be free of anaplasmosis, including Switzerland, warranted studies on the role of putative tick vector(s) [17]. The broad distribution range of *D. reticulatus*, which extends from the British isles to Central Asia [18], as well as the expanded geographic distribution of this tick as recently reported in Germany [19], Hungary [20] and the Netherlands [21], warrants further study of *D. reticulatus *as a vector for *A. marginale *in Europe.

## Results

### Infection and acquisition feeding

**Figure 1:**
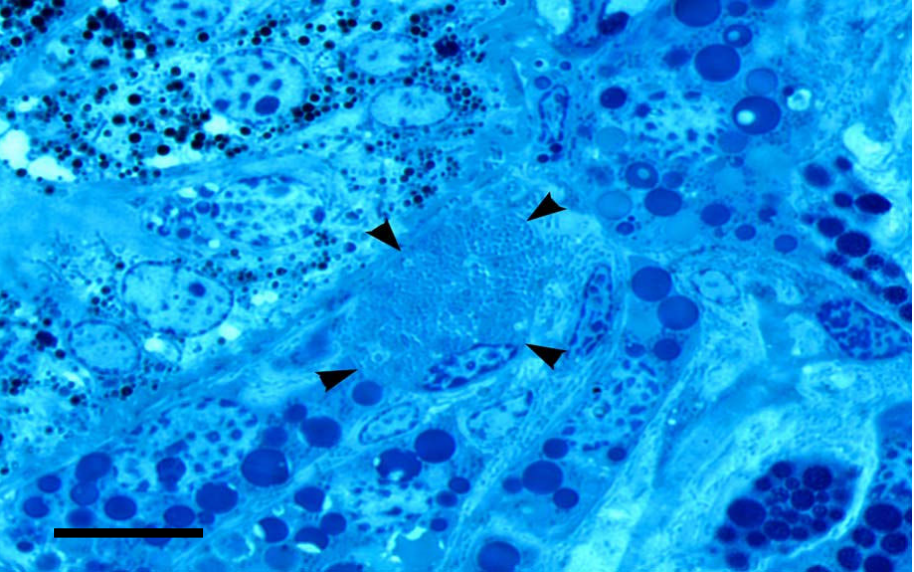
Light micrograph of male *D. reticulatus *salivary gland cell containing several *A. marginale *colonies (arrowheads). Bar = 10 μm.

Infection of calf No. 4291 with the *A. marginale *Zaria isolate was detected on day 20 post exposure (PI) when the body temperature increased to 39.9°C and depression and anorexia were observed. The percent reduction PCV was 50% and the *A. marginale *percent parasitized erythrocytes (PPE) was 6% (Table 1). *A. marginale *infection was subsequently confirmed by *msp4 *PCR. After infestations of the calf on the day 34 p.i. with 80 male and 5 female *D. reticulatus *ticks when the PPE was 0.6% (minimum 1000 erythrocytes counted), all female ticks and 66 of the male ticks attached and fed successfully. Based on PCR testing of one salivary gland from each of the 30 male tick halves, the infection percentage was 30%. The presence of *A. marginale *colonies in salivary gland cells was confirmed by light microscopy examination (Figure 1) in the other half of the PCR positive ticks.

**Table 1:** Clinical findings of acquisition and transmission feeding of *D. reticulatus *ticks.

**Calf number**	**Number of ticks/feeding (days)**	**Incubation period (days)^c^**	**Maximum temperature (°C)**	**PCV reduction (%)**	**Maximum parasitemia (%)**
4291^a^	80/7	20	39.9	50	6
9191^b^	30/7	35	39.9	37.5	2

^a ^Infected intravenously with *A. marginale *(Zaria isolate) blood stabilate.

^b ^Infested with *D. reticulatus *adult males fed on calf 4291.

^c ^Number of days to first observation of infected blood cells on a stained blood smears.

### Transmission feeding

Male *D. reticulatus *ticks transmitted *A. marginale *Zaria isolate to calf No. 9191. On day 27 p.i. the calf tested PCR positive. Inclusion bodies were detected in erythrocytes on day 35 in Giemsa-stained blood smears and the peak parasitemia was 2%. While minimal clinical symptoms of anaplasmosis were observed, the body temperature increased during the peak of parasitemia up to 39.9°C, the percent reduction PCV was 37.5% (Table 1) and the mucosal membranes became pale. Five out of 27 (18.5%) ticks that were attached successfully were PCR positive for *A. marginale*.

### Verification of isolate identity

**Figure 2:**
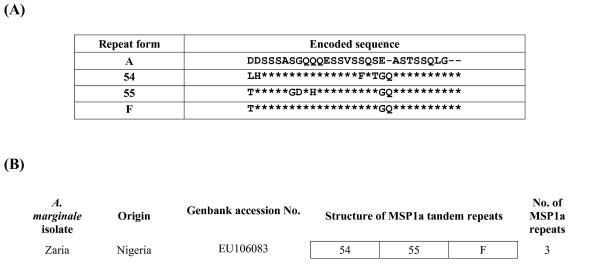
Sequence and structure of MSP1a tandem repeats in the Zaria isolate of *A. marginale*. (A) The one letter amino acid code was used to depict the different sequences found in MSP1a repeats. Asterisks indicate identical amino acids and gaps indicate deletions/insertions with respect to the reference repeat A. (B) The structure of the MSP1a repeats region was represented using the repeat forms described in (A). Description of MSP1a repeats was updated after de la Fuente et al. [22].

The *A. marginale *isolate genetic identity was confirmed by PCR in samples collected during persistent infection in calf, during replication and development in ticks and after subsequent transmission to the susceptible calf. Subsequent sequencing of the *msp4 *and *msp1α *genes also confirmed the isolate identity since the *msp4 *and *msp1α *sequences were the same in all tick and cattle samples. The *msp1α *sequence of the Zaria isolate contained two novel repeat forms which were labelled as 54 and 55 following the nomenclature of de la Fuente *et al.*[22] (Figure 2).

#### Sequence accession numbers

The GenBank accession numbers for msp1α and msp4 sequences of the Zaria isolate of *A. marginale *are [GenBank: EU106083] and [GenBank: EU106082] respectively.

## Discussion

*A. marginale *infection in cattle and wild ruminants was reported in several parts of Europe, including the Mediterranean countries of Spain and Portugal [6,7], Italy [4,5], and occasionally in France [23], the Alpine region of Switzerland [8,17] and more recently in Hungary [10]. However, the epidemiology of anaplasmosis in Europe has not been thoroughly investigated and local vector species were not identified.

In the Mediterranean region, several tick species have been incriminated as the potential biological vectors of *A. marginale*. *Hyalomma m. marginatum *and *Rhipicephalus bursa *were found on Iberian red deer in Spain that proved to be infected with *A. marginale *[7] and these ticks were therefore listed as putative tick vectors. Furthermore, *A. marginale *is endemic in Sicily and has been reported elsewhere from Italy, and several tick species have been identified in this area that may be vectors, including *Rhipicephalus turanicus *and *Haemaphysalis punctata *that were collected from *A. marginale *infected cattle [5].

Although anaplasmosis was reported in more northern latitudes, the tick vectors have not been identified. For instance, *A. marginale *was recently reported to be endemic in Hungary and the predominant tick species present on cattle in the study area was *D. reticulatus *[10]. In the United States, several *Dermacentor *spp. (*D. variabilis*, *D. andersoni *and *D. albipictus*)are known to be vectors of anaplasmosis [14]. While *D. reticulatus *is known to be a vector of *Babesia canis *[24], *Rickettsia slovaca *[25] and *Coxiella burnetii *[26,27], the vector competency of this tick for *A. marginale *has not been determined until now.

The *A. marginale *strain used in this study originated from Zaria, Nigeria. Although bovine anaplasmosis occurs in large areas of Africa and a few isolates from South Africa have been characterized, this isolate from West Africa had not been reported previously and proved to have unique *msp1a *and *msp4 *sequences. The *A. marginale msp4 *gene, which is a stable marker for the genetic characterization of strains, does not undergo antigenic variation when cycling between tick and mammalian hosts [28]. MSP1a, encoded by *msp1α*, is involved in the adhesion and transmission of *A. marginale *by ticks and varies in the number and sequence of amino-terminal tandem repeats among geographic strains [22].

## Conclusion

In this research we have demonstrated the vector competency of male *D. reticulatus *as an experimental biological vector of *A. marginale *by intrastadial transmission. Further studies are needed to confirm the vectorial role of *D. reticulatus *in Europe by use of *A. marginale *isolates derived from naturally infected cattle and ticks from endemic areas. *Dermacentor *spp. from *A. marginale *endemic regions should be studied, including *D. marginatus *which is commonly found on cattle and wildlife reservoir hosts.

## Methods

### Experimental animals

Two Holstein-Friesian calves, 8 months of age (No. 4291 and No. 9191), were used. Both animals had no previous exposure to ticks and were confirmed to be *A. marginale *free by examination of Giemsa-stained blood smears and *msp4 *PCR. All the ticks feeding and *A. marginale *infection were approved by the Animal Experiments Committee (DEC) of the Faculty of Veterinary Medicine, Utrecht University (DEC No. 0604.0801). Both animals were euthanized at the end of the experiment.

### *A. marginale *isolate

A Nigerian *A. marginale *isolate used for these studies was obtained from a naturally infected bovine from Zaria, Nigeria in 1986. This isolate was subsequently passaged in splenectomized calves, and blood samples were collected at the peak of parasitemia, prepared with 10% DMSO as stabilate and stored in 2 ml aliquots in liquid nitrogen.

### Ticks

Adult *D. reticulatus *ticks were collected during October 2006. by dragging vegetation in the area of the Dintelse Gorzen, The Netherlands. The absence of *A. marginale *infection in collected ticks was confirmed in 344 randomly selected ticks by use of an *A. marginale *specific PCR followed by reverse line blot hybridization (RLB) [21]. The ticks were maintained in the laboratory at 20°C/90% relative humidity. Male ticks, allowed to acquire infection by feeding on an infected calf, were used for these studies because of their putative role in transmission of *A. marginale *[15,16].

### Infection of ticks

For infection of calves, the *A. marginale *blood stabilate was thawed and inoculated intravenously (IV) into the jugular vein of an eight-month old non-splenectomized and tick-naïve Holstein-Friesian calf (No. 4291). Rectal temperature was measured and registered daily and calf was observed for anemia and other signs consistent with anaplasmosis. Giemsa-stained blood smears were made and examined daily during the acute stage of the infection and twice weekly during the persistent stage of the infection. The packed cell volume (PCV) was determined using the microhematocrit technique. On day 34 p.i., 5 female and 80 male *D. reticulatus *ticks were placed in cotton patches glued to shaved area on the back of the calf. The ticks were allowed to acquisition feed for 7 days, after which the engorged females were removed and discarded and the fed male ticks were placed in an incubator at 20°C with 90% relative humidity and a 12:12 h photoperiod for 7 days. This holding period provided time for the development and multiplication of *A. marginale *in tick midguts and other tissues [29]. Thirty male ticks were randomly selected and cut in half with a razor blade separating the right and left sides. The salivary gland from one tick half was dissected for PCR testing, while the other tick half was fixed for light microscopy studies.

### Transmission feeding

A second eight-month old, tick-naïve and non-splenectomized Holstein-Friesian calf (No. 9191) was used for the transmission feeding of *D. reticulatus*. A group of 30 acquisition fed male *D. reticulatus *ticks was allowed to feed a second time for 7 days on this calf. After transmission feeding, ticks were removed and the salivary glands from one half of each tick were dissected for subsequent *msp4 *PCR testing. Body temperature was recorded daily and calf observed closely for the signs of illness. Blood samples were collected from the calf for determination the percent reduction PCV using the microhematocrit technique and for the preparation of Giemsa-stained blood smears. DNA was extracted from the blood samples and tested for the presence of *A. marginale *by the *msp4 *PCR [22].

### Light microscopy studies

For light microscopy studies, a half of each tick was fixed in 2% glutaraldehyde in O.2 M sodium cacodylate buffer. The halves were then post fixed in osmium tetraoxide in O.2 M sodium cacodylate buffer, dehydrated in graded series of ethanol (70% – 100%) and embedded in epoxy resin. Thick sections (1.0 μm) were cut and stained with Malory's stain for observation with a light microscope. Light micrographs were recorded with Leica DM LB with Spotcam camera system (Oklahoma State University, Stillwater, OK, USA).

### Molecular Diagnostics

DNA was extracted from 200 μl of blood and from individual tick salivary glands using NucleoSpin DNA extraction kit (Macherey-Nagel, Düren, Germany) following the manufacturer's protocol for the purification of genomic DNA from blood and insects. The DNA was eluted with water and stored at -20°C. A PCR assay amplifying the *A. marginale msp4 *gene was performed on blood samples of the two calves used for tick feeding and *D. reticulatus *salivary gland DNA samples, followed by sequencing as described previously [30].

The *msp1α *gene was amplified from DNA extracted from the blood of *A. marginale *infected calves and tick salivary glands as described previously [22], but using forward primer **MSP1aATG**: 5'-TGTGTGTGTGTTATGT-3' instead of primer MSP1aP. Amplified and column purified samples were cloned in the pGEM-T vector (Promega) following the manufacturer's protocol and used directly for sequencing (Secugen SL, Madrid, Spain). The resulting *msp1α *and *msp4 *gene sequences were compared to sequence data available from GenBank using the BLAST 2.2.15 program [31]. Multiple sequence alignment was performed using the program Align X (Vector NTI Suite V5.5., Invitrogen, North Bethesda, MD USA) with an engine based on the Clustal W algorithm [32].

## Authors' contributions

ZZ carried out most of experiments and drafted the manuscript. AN contributed to the design of the study and acquisition of the data and helped with drafting the manuscript. JF contributed to acquisition of molecular biology data and helped with drafting and revision of the manuscript. KK performed the light microscopy study and helped with drafting and revision of the manuscript. FJ participated in design and coordination of the study and helped with drafting and revision of the manuscript. All authors read and approved the final manuscript.
